# The Renal Protective Effect of Jiangya Tongluo Formula, through Regulation of Adrenomedullin and Angiotensin II, in Rats with Hypertensive Nephrosclerosis

**DOI:** 10.1155/2015/428106

**Published:** 2015-10-18

**Authors:** Lin Han, Yan Ma, Jian-guo Qin, Li-na Li, Yu-shan Gao, Xiao-yu Zhang, Yi Guo, Lin-mei Song, Yan-ni Luo, Xiao-yi Chi

**Affiliations:** ^1^College of Basic Medicine, Beijing University of Chinese Medicine, Beijing 100029, China; ^2^Molecular Research in Traditional Chinese Medicine Group, Department of Pathophysiology and Allergy Research, Center of Pathophysiology, Infectiology & Immunology, Vienna General Hospital, Medical University of Vienna, Waehringer Guertel 18-20, 1090 Vienna, Austria; ^3^Department of Nephrology, Dongfang Hospital, The Second Clinical Medical College of Beijing University of Chinese Medicine, Beijing 100078, China

## Abstract

We investigated the effect of Jiangya Tongluo (JYTL) formula on renal function in rats with hypertensive nephrosclerosis. A total of 21 spontaneously hypertensive rats (SHRs) were randomized into 3 groups: valsartan (10 mg/kg/d valsartan), JYTL (14.2 g/kg/d JYTL), and a model group (5 mL/kg/d distilled water); Wistar Kyoto rats comprised the control group (*n* = 7, 5 mL/kg/d distilled water). Treatments were administered by gavage every day for 8 weeks. Blood pressure, 24-h urine protein, pathological changes in the kidney, serum creatinine, and blood urea nitrogen (BUN) levels were estimated. The contents of adrenomedullin (ADM) and angiotensin II (Ang II) in both the kidney and plasma were evaluated. JYTL lowered BP, 24-h urine protein, serum creatinine, and BUN. ADM content in kidneys increased and negatively correlated with BP, while Ang II decreased and negatively correlated with ADM, but there was no statistically significant difference of plasma ADM between the model and the treatment groups. Possibly, activated intrarenal renin-angiotensin system (RAS) plays an important role in hypertensive nephrosclerosis and the protective function of ADM via local paracrine. JYTL may upregulate endogenous ADM level in the kidneys and antagonize Ang II during vascular injury by dilating renal blood vessels.

## 1. Introduction

Hypertensive nephrosclerosis is one of the most common and serious chronic complications in primary hypertension. It is an independent risk factor for end-stage renal disease (ESRD) [1]; approximately 25% of patients with hypertensive nephrosclerosis require a kidney transplant [2]. Data from the United States Renal Data System suggest that hypertension is the second cause of ESRD and the multiple risk factor intervention study revealed that approximately 49% of ESRD cases are caused by hypertension. By 2002, there were 160 million patients with hypertension in China and approximately 9.6% of patients were on dialysis as a result of renal artery sclerosis due to hypertension. Improvements in the early prevention and treatment of hypertension have resulted in significant reductions in mortality from cardiovascular complications caused by hypertension; however, for hypertensive nephrosclerosis, the mortality rates have not significantly improved [2]. Therefore, research efforts are now being focused on the mechanism and prevention of hypertensive nephrosclerosis. Currently, the treatment of hypertensive nephrosclerosis involves controlling blood pressure and preglomerular arteriolar resistance, reducing intraglomerular pressure, and improving renal ischemia. However, this approach has failed to show a curative effect in approximately 50% of patients and the application of angiotensin-converting enzyme inhibitor (ACEI) drugs is limited when renal function is impaired beyond a specific threshold (serum creatinine >3 mg/dL). Therefore, further research on new drugs and treatment approaches to delay the progression of hypertensive nephrosclerosis towards end-stage renal failure is required.

Adrenomedullin (ADM) is a vasodilator peptide that was originally isolated from the extract of human pheochromocytoma in 1993. It has numerous biological effects and is present in a variety of tissues and organs, particularly in the heart, kidney, and lungs [3]. ADM is mainly produced by vascular endothelial and smooth muscle cells, while mesangial and epithelial cells also secrete ADM [4]. When combined with calcitonin receptor-like receptors (CRLR) and receptor activity-modifying proteins 2 and 3 (RAMP2 and RAMP3), ADM has a strong vasodilator effect [5]; it increases renal blood flow and glomerular filtration via the expansion of efferent and afferent arterioles [4]. ADM exerts important vascular and renal protective effects through its interaction with Ang II and other vasoconstrictive substances [6]. Therefore, ADM is considered important in preventing high blood pressure and hypertension-induced organ damage. Consequently, investigation of the potential renal protective effects of ADM has become a research focus in the field of hypertension nephrosclerosis.

We routinely use Jiangya Tongluo (JYTL) at our daily clinic to treat hypertensive nephrosclerosis and we have observed good clinical curative effect. To gain insight into the mechanisms of regulation of JYTL in hypertensive nephrosclerosis, we investigated the effects of JYTL on blood pressure, renal function, and the expression of ADM and Ang II in kidney and plasma using a spontaneous hypertensive rat (SHR) experimental model to explore its decompression and protective effects on kidney.

## 2. Materials and Methods

### 2.1. Animal Models and Drugs

Sixteen-week-old, male SHRs (mean weight = 200 ± 10 g) were purchased from Vital River Laboratories (Beijing, China; number SCXK, 2002-2003). The animals were housed in the Central Laboratory at Beijing University of Chinese Medicine, with a 12-h light/dark cycle and free access to food and water. The SHRs were fed a normal diet for a week and were then randomized to one of 3 groups: model (5 mL/kg/d distilled water by gavage), valsartan (10 mg/kg/d valsartan by gavage), and JYTL (14.2 g/kg/d JYTL by gavage). The control group comprised Wistar Kyoto rats (5 mL/kg/d distilled water by gavage). These doses were determined from a previous pharmacodynamic experimental study [7]. Each group was then sacrificed after an 8-week treatment period. All experimental procedures were conducted in accordance with the guidelines for the use of experimental animals and approval was granted by the Institutional Review Committee on Animal Care and Use at the Experimental Animal Centre at Beijing University of Chinese Medicine (Certificate of Conformity: SCXK, Beijing, 2012-0001).

#### 2.1.1. Preparation of JYTL Decoction

JYTL is frequently used in clinical practice for treatment of hypertension and is composed of 30 g* Nacre* (Zhen Zhu Mu, ZZM), 15 g* Cassia occidentalis* (Cao Jue Ming, CJM), 12 g* Safflower* (Hong Hua, HH), 20 g* Salvia miltiorrhiza* (danshen, DS), and 15 g* Chrysanthemum* (juhua, JH). The medicinal herbs were provided by Pharmacy Department at Dongfang Hospital, Beijing, China. The herbs were first cut and boiled together in 6x volume of water for 0.5 h (first extraction). Residue from the first extraction was boiled in 8x volume of water for 25 min. Finally, the filtered solutions were combined and concentrated into an aqueous extract containing 1.2 g/mL raw herbs. Valsartan was purchased from Novartis Pharma Ltd (Beijing, China).

### 2.2. Blood Pressure Detection

Systolic blood pressure (BP) was monitored prior to treatment (0 weeks) and after 2, 4, 6, and 8 weeks of treatment using a noninvasive, computerized, tail-cuff system (BESN-II, Desai Production Biotechnology Co, Nanjing, China) and performed by specifically assigned investigators at regular intervals in order to minimize error. The mean value from 3 measurements of BP was taken for each rate, representing the sample systolic pressure.

### 2.3. Pathological Examination of Renal Tissue

After 8 weeks of treatment, rats were sacrificed under chloral hydrate anesthesia (3.5 g/kg administered via intraperitoneal injection). Blood was rapidly sampled by abdominal aorta puncture and the serum was stored at −80°C prior to analysis. Renal tissue was excised, washed in physiological saline, snap-frozen in liquid nitrogen, and then stored at −80°C. Kidney tissues were fixed with 4% paraformaldehyde, embedded in paraffin, and cut into 3-*μ*m thick sections. Hematoxylin-eosin (HE) staining was performed in order to assess glomerular and vascular injuries and perivascular lesions such as fibrosis under an electronic scanner (SIP NO. MIC 01579, Zeiss Co, Germany).

### 2.4. Determination of Urinary Protein over 24 Hours

Prior to and after 8 weeks of treatment, the rats were placed in metabolic cages where food was not provided (but water was available). Urine samples were collected over 24 h to calculate the total urinary output and 5 mL was collected for centrifugation; the supernatant was stored at −20°C. A commercial radioimmunoassay (RIA) kit (PLA Institute of RIA, Beijing, China) was used to estimate urine protein over 24 h, in accordance with the manufacturer's protocol.

### 2.5. Determination of Serum Creatinine and Blood Urea Nitrogen Levels

After 8 weeks of treatment, blood was collected from the abdominal aorta and serum was separated by centrifugation (1500 ×g) at 4°C for 10 min and stored in Eppendorf tubes. Blood urea nitrogen (BUN) and creatinine levels were determined by the Jaffe and diacetyl-oxime methods, in accordance with the manufacturer protocol.

### 2.6. Determination of Ang II and ADM Content in the Kidney and Plasma

After 8 weeks of treatment, rats were sacrificed and blood was sampled from the abdominal aorta. Fresh kidney tissues were collected, weighed, quickly ground with normal saline, and boiled in water at 100°C for 10 min. The homogenate was centrifuged (1500 ×g) at 4°C for 15 min and the supernatant was preserved at −20°C. Ang II and ADM content in the renal tissue and plasma was determined using an RIA Kit (PLA Institute of RIA, Beijing, China) in accordance with the manufacturer's protocol. The protein content of the supernatant was also simultaneously determined for correction.

### 2.7. Statistical Analysis

All data were analyzed using SPSS 16.0 software (SPSS Inc., Chicago, USA). Statistical significance was analyzed using a one-way analysis of variance, followed by the post hoc Student-Newman-Keuls multiple comparison test. All data are expressed as mean ± standard error of the mean (SEM) and *P* < 0.05 was considered statistically significant.

## 3. Results

### 3.1. Effect of JYTL on Blood Pressure

**Figure 1:**
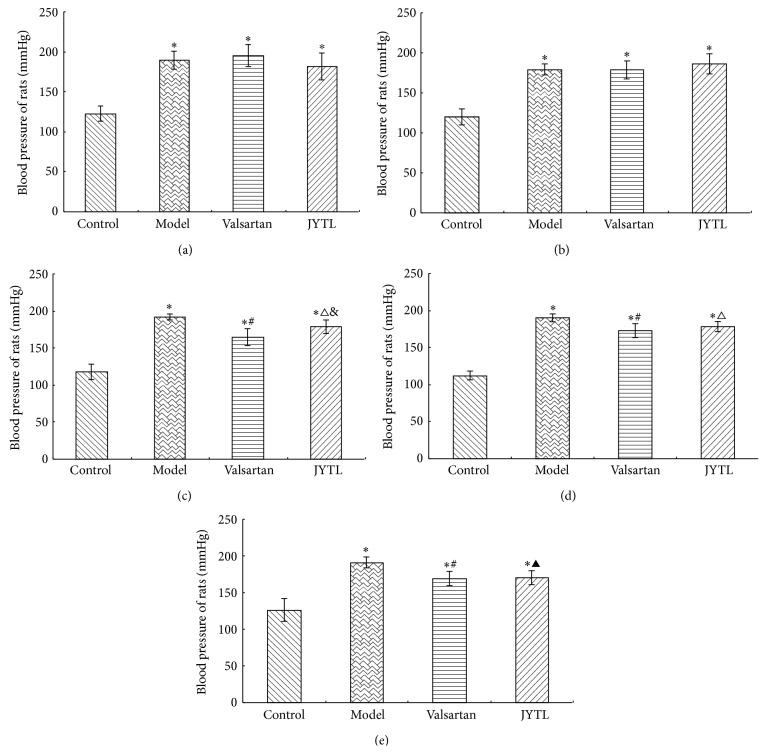
Effect of JYTL on mean ± SEM blood pressure (*n* = 7 per study group). (a) Before treatment. (b) After 2 weeks of treatment. (c) After 4 weeks of treatment. (d) After 6 weeks of treatment. (e) After 8 weeks of treatment. ^∗^ *P* < 0.01, model, valsartan, and JYTL rats versus control rats. ^#^ *P* < 0.01, rats in the valsartan group versus model rats. ^△^ *P* < 0.05 and ^▲^ *P* < 0.01, rats in the JYTL group versus model rats. ^&^ *P* < 0.05, rats in the JYTL group versus valsartan group.

Animal models in this study comprised SHRs with knocked out genes. Prior to treatment, the BP of rats in the model group and both treatment groups was significantly higher than in the control group (*P* < 0.01) (Figure 1(a)); the model and treatment groups were comparable indicating that the model was successfully established. No decrease in BP of rats was observed in the valsartan and JYTL groups compared with the model group after 2 weeks of treatment (Figure 1(b)). However, at 4, 6, and 8 weeks of treatment, the BP of rats in the JYTL and valsartan groups was significantly lower than that in the model group (*P* < 0.01 and *P* < 0.05). Furthermore, at 4 weeks, the BP of rats in the valsartan group was lower than that in the JYTL group (Figure 1(c)), while at 6 and 8 weeks, there was no statistically significant differences between the two groups (Figures 1(d) and 1(e)).

### 3.2. Effects of JYTL on Renal Pathological Morphology

**Figure 2:**
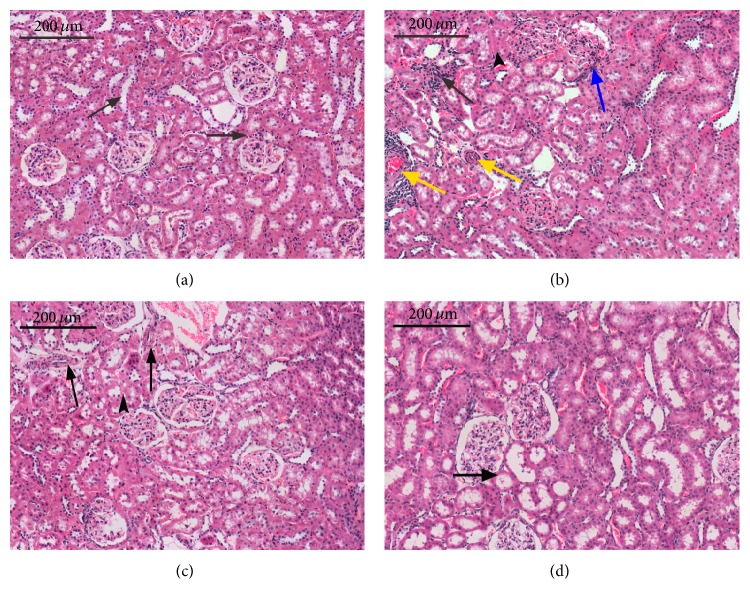
Histologic studies of JYTL in hypertensive nephrosclerosis rats after HE staining. (a) Control group. (b) Model group. (c) Valsartan group. (d) JYTL group. Bar = 200 *μ*m; same magnification for all panels. (a) The arrows point to the glomerular and the lined epithelia of renal tubules in normal condition. (b) Glomerular ischemia and sclerosis (blue arrow), interlobular artery with marked myointimal proliferation (yellow arrows), tubular atrophy and hyaline degeneration (little black arrow), and interstitial fibrosis with inflammatory cells hyperplasia (black arrow). (c) Interlobular artery mild-thickening with tubular vacuolar degeneration. (d) Tubular ectasia with hyaline degeneration. HE in 3-*μ*m thick sections, 200.

We observed severely pathological lesion in SHRs by hematoxylin-eosin staining. It comprised glomerular ischemia and sclerosis, tubular atrophy and hyaline degeneration, and interstitial fibrosis with inflammatory cells hyperplasia, and the interlobular arteries show intimal thickening (Figure 2(b)). Such lesions were considerably diminished by JYTL and valsartan; the pathological change represented interlobular artery mild-thickening with tubular vacuolar degeneration (Figure 2(c)) and tubular ectasia with hyaline degeneration (Figure 2(d)). Furthermore, the renal structure of Wistar Kyoto rats appeared normal (Figure 2(a)). This indicated that JYTL and valsartan could significantly protect the kidney from hypertension-induced lesions. This result strongly supported the therapeutic potential of JYTL against hypertensive nephrosclerosis.

### 3.3. Effects of JYTL on Urinary Protein Quantity over 24 Hours

**Figure 3:**
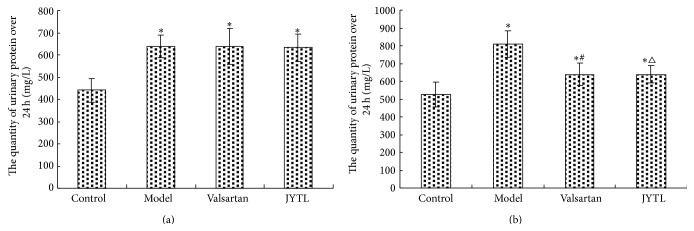
Effect of JYTL on the mean ± SEM urine protein content over 24 h (*n* = 7 per study group). (a) Before treatment. (b) After 8 weeks of treatment with JYTL. ^∗^ *P* < 0.01, model, valsartan, and JYTL rats versus control rats. ^#^ *P* < 0.01, rats in the valsartan group versus model rats. ^△^ *P* < 0.01, rats in the JYTL group versus model rats.

The quantity of urinary protein over 24 h was statistically significantly higher in the model group and both treatment groups compared with the control group (*P* < 0.01); there was no statistically significant difference between model and treatment groups, indicating a successful model. After 8 weeks of treatment, the 24-h urine protein level was significantly reduced in both the model and treatment groups (*P* < 0.01); it was slightly lower in the JYTL group than in the valsartan group; however, these differences were not statistically significant (Figures 3(a) and 3(b)).

### 3.4. Effects of JYTL on Renal Function

**Figure 4:**
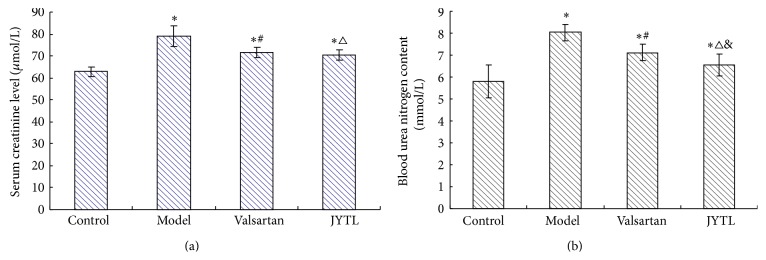
Effect of JYTL on mean ± SEM renal function (*n* = 7 in each group). (a) Serum creatinine. (b) Blood urea nitrogen. ^∗^ *P* < 0.01, model, valsartan, and JYTL rats versus control rats. ^#^ *P* < 0.01, rats in the valsartan group versus model rats. ^△^ *P* < 0.01, rats in the JYTL group versus model rats. ^&^ *P* < 0.05, JYTL versus valsartan group.

Following 8 weeks of treatment, serum creatinine levels were significantly reduced in both JYTL and valsartan groups (*P* < 0.01); serum creatinine levels in JYTL group were slightly lower than in the valsartan group; however there was no statistically significant difference between the two groups (*P* > 0.05). The blood urea nitrogen content was significantly reduced in both JYTL and valsartan groups after the 8-week treatment period (*P* < 0.01). While BUN was lower in the JYTL group than in the valsartan group, this difference was statistically significant (*P* < 0.05) (Figures 4(a) and 4(b)).

### 3.5. Effects of JYTL on the Expression of Ang II and ADM in the Kidneys and ADM in Plasma

**Figure 5:**
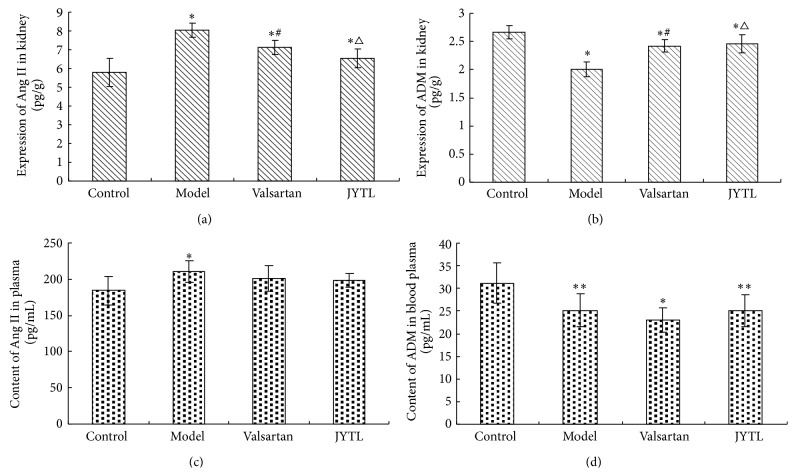
Effect of JYTL on mean ± SEM ADM and Ang II content (*n* = 7 per group). (a) Ang II in kidney. (b) ADM in kidney. (c) Ang II in plasma. (d) ADM in plasma. ^∗^ *P* < 0.01 and ^∗∗^ *P* < 0.05, model, valsartan, and JYTL rats versus control rats. ^#^ *P* < 0.01, rats in the valsartan group versus model rats. ^△^ *P* < 0.01, JYTL group versus model group.

The results of RIA showed that kidney Ang II expression in the model, JYTL, and valsartan groups was statistically significantly higher than that in the control group (*P* < 0.01). Ang II expression was statistically significantly lower in the valsartan and JYTL groups than in the model group (*P* < 0.01). While Ang II expression in valsartan group was slightly higher than the JYTL group, these differences were not statistically significantly different (Figure 5(a)). Levels of ADM in the model, JYTL, and valsartan kidneys were statistically significantly lower than the control (*P* < 0.01) and significantly higher in the valsartan group compared with the model (*P* < 0.01). While the level of ADM in the valsartan group was slightly higher than that in the JYTL group, this difference was not statistically significant (Figure 5(b)). After 8 weeks of treatment, the content of Ang II in plasma in the model group was statistically significantly higher than that in the control group (*P* < 0.01). In treatment groups though the levels of Ang II were higher than that of control group, but there was no statistically significant difference between them and also there was no statistically significant difference between the model and treatment groups (Figure 5(c)). Meanwhile, the content of ADM in the plasma in the model group and both treatment groups was statistically significantly lower than that in the control group (*P* < 0.01 and *P* < 0.05, resp.), but there was no statistically significant difference between the model and treatment groups (Figure 5(d)).

### 3.6. Correlation of ADM with Ang II and BP

**Figure 6:**
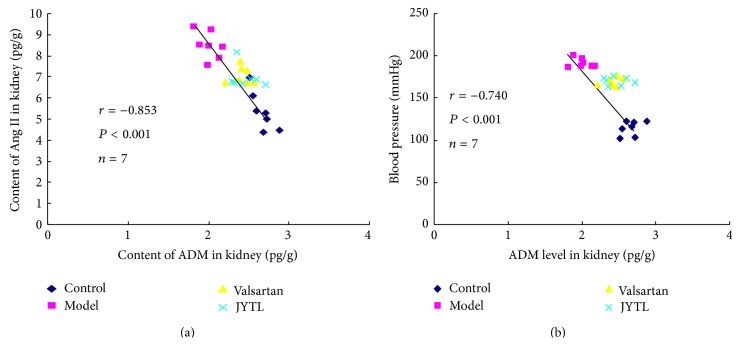
(a) Correlation between ADM and Ang II levels in the kidney. (b) Correlation between BP and ADM levels in the kidney.

There was a statistically significant negative correlation between ADM and Ang II and between ADM and BP in the kidneys (Figures 6(a) and 6(b)).

## 4. Discussion

Hypertensive nephrosclerosis is a form of hypertension-induced arteriolar nephrosclerosis and it results from benign arteriolar nephrosclerosis. Pathological changes indicative of the condition include renal afferent arteriole hyaline degeneration and myointimal hypertrophy in the arteria interlobulares and arteria arcuata [8, 9]. These lead to ischemic changes in the glomeruli and interstitium and consequently compromise renal function [10].

Decompression is the modern, universally accepted form of hypertensive nephrosclerosis therapy. Studies have demonstrated a significant contribution of ACE I and Ang II type 1 receptor blockers (ARB) in the prevention of renal and cardiovascular damage [11, 12]. However, this form of treatment has failed to demonstrate an obvious curative effect in approximately 50% of patients. Furthermore, the vasodilator effect of ACEI and ARB on efferent arterioles is superior to afferent arterioles that impacts the compensatory mechanisms of intraglomerular pressure, hypertransfusion, and hyperfiltration and accelerates renal function aggravation. For those patients with renal insufficiency, administering ACEI frequently predisposes them to hyperkalemia. Therefore, when renal functional impairment crosses a specific threshold (serum creatinine >3 mg/dL), the application of ACE I and ARB is limited. Consequently, it has become important to explore new therapeutic methods to delay the progression of hypertensive nephrosclerosis towards end-stage renal disease.

The pathogenesis and etiology of hypertensive nephrosclerosis are complex. The mechanism behind the condition is as follows. (1) Glomeruli hypertension results in vascular endothelial cell damage and increases vasoconstrictor (Ang II, ET-1) and platelet derived growth factors and the synthesis and secretion of extracellular matrix [11, 12]. (2) Elevations in the glomerular capillary pressure lead to an increase in some of the transforming growth factors such as TGF-*β*1; this procession stimulates collagen deposition and the proliferation of mesangial cells and increases the extracellular matrix, eventually leading to kidney sclerosis [13]. (3) The glomerular ischemia inflammatory response causes increases in vascular injury, vasoactive substances, chemical chemokines, and mitogenic factors and can aggravate kidney damage [14]. Reactive oxygen species can stimulate enzymes which have oxidative stress sensitivity and nuclear transcription factors (NF-*κ*B and AP-1) and can increase the expression levels of cytokine and chemotactic and adhesion factors. The final stages of the mechanism of hypertensive nephrosclerosis include triggering renal interstitial inflammation, renal fibroblasts proliferation and conversion, and the formation of renal fibrosis [15].

The functional impairment of vascular endothelial cells therefore plays a critical role in the pathogenesis of hypertensive nephrosclerosis. Apoptosis of microvascular endothelial cells induces microvessel rarefaction and increases peripheral resistance, which in turn increases BP, decreases the ability of substance and energy metabolism, and reduces reserve capacity and material exchange ability, resulting in the injury of target organs. This present study found that Ang II promoted endothelial cell apoptosis in a concentration-dependent manner. Endothelial cell apoptosis induces an imbalance of proliferation versus apoptosis of smooth muscle cells. This procession eventually results in changes to the structure and function of blood vessels and target organ damage [16–18]. Therefore, inhibition of blood vessel injuries by Ang II is key to the treatment of hypertension-induced organ injury.

AMD is a novel vasorelaxant peptide. It is critical for both vascular remodeling and blood pressure regulation and plays an important role in the protection of kidneys and blood vessels [5]. It dilates the blood vessels in two ways: endothelium-dependent dilation and endothelium-independent dilation [19–21]. ADM can increase blood flow and glomerular filtration rate through the dilation of the afferent and efferent arterioles [4]. It can also block the action of Ang II-induced mitogen-activated protein kinase, resulting in the prohibition of overproliferation of smooth muscle cells [4, 22] and the inhibition of endothelial cell apoptosis through a NO-dependent mechanism [23]. ADM promotes impaired endothelial cells reendothelialization through the cyclic adenosine monophosphate and phosphatidylinositol 3-kinase pathways, consequently playing a role in vascular endothelial growth and the promotion of angiogenesis [24, 25].

Collagen and elastin, which are secreted by fibroblasts and inhibit vascular sclerosis through the regulation of matrix metalloproteinase-2 activity and protein expression, are reduced by ADM [26]. Therefore, ADM is regarded as a crucial factor not only for preventing an increase in BP but also for preventing target organ damage. Consequently, studies of ADM have become a research focus in the field of hypertension nephrosclerosis. ADM is an endogenic substance involved in a variety of bodily functions in an autocrine and paracrine manner; however, it is expensive and therefore unsuitable for long-term therapy. Therefore, the development of drugs that promote the generation of endogenous ADM and improve its bioactivity has become an urgent requirement.

Traditional Chinese medicine (TCM) has been used since a long time and positive treatment effects have been observed in hypertensive nephrosclerosis [27, 28]. JYTL formula used in this study is composed of* Nacre, Cassia occidentalis, Safflower, Salvia miltiorrhiza, and Chrysanthemum*. This preparation was derived from the JYTM formula invented by Shikui Guo (one of the most prestigious TCM cardiovascular specialists) and it has obvious therapeutic effects on cardiovascular disease [29]. Modern pharmacological research has shown that* Nacre* exerts an anti-inflammatory effect in the treatment of vascular headache.* Cassia occidentalis* depresses blood pressure, reduces the extracellular matrix, inhibits mesangial cell proliferation, reduces proteinuria, and improves renal function.* Safflower and Salvia miltiorrhiza* have antioxidant and anti-inflammatory effects; clinical validation confirms their curative effect in terms of promoting blood circulation to remove blood stasis. They are also known to play a major role in renal interstitial fibrosis, vascular endothelial injury, and oxidative stress [29–33].* Chrysanthemum* can cause significant dilation of the coronary artery, increase in coronary blood flow, and improvement of myocardial cells for hypoxia tolerance. It also plays an important role in decompression and often synergizes with other drugs to reduce hypertension [34]. In this study, we used JYTL to treat hypertensive nephrosclerosis in a rat model and explore the mechanisms underlying its decompression and protective effects on kidney function.

The results demonstrated that after 4 weeks of treatment with JYTL, the BP of rats decreased, though valsartan group showed better results than the JYTL group at this time. However, with prolonged JYTL treatment, at 6 and 8 weeks, JYTL group showed similar antihypertensive effect as valsartan. Since there are no obvious medical contraindications to TCM, JYTL also can be used when renal functional impairment crosses a specific threshold (serum creatinine >3 mg/dL). On the other hand, valsartan causes reduced glomerular filtration rate (GFR), aggravates renal injury, and is not recommended to use under such conditions. Our results also showed that JYTL reduced albuminuria, improved renal function, and alleviated renal pathological damage in SHR. To further explore the mechanism of action of JYTL, we estimated the content of Ang II and ADM in the kidneys and plasma. We observed that the plasma ADM content decreased rapidly in the 24-week-old SHR which is different from previous research [35, 36]. This may be due to progressive endothelial cell injury and the self-regulation function in a state of decompensation. However, valsartan and JYTL showed no effect on the level of ADM and Ang II in plasma, which is generally comparable to previous studies [37, 38]. Some authors have described high levels of Ang II in kidney tissue [39] and locally generated Ang II has been implicated in paracrine regulatory mechanisms, leading to altered proliferative and synthetic responses of cells [40]. Therefore, we speculated that the renal protective effect of ADM is not associated with circulating ADM and perhaps renal local RAS plays a more important role in the process of hypertensive nephrosclerosis. We explored the correlation between ADM and Ang II in the kidneys. The data showed that ADM content in SHR kidneys decreased significantly as compared to that in control rats, while Ang II increased. ADM protein content showed a statistically significant, negative correlation between BP and Ang II levels. JYTL and valsartan upregulated the level of ADM and downregulated Ang II level in the kidney. Thus, it can be concluded that ADM may act as a paracrine factor affecting renal function during hypertensive nephrosclerosis and the renal protective effect of JYTL may occur via upregulation of endogenous ADM levels in the kidney and antagonism of vascular injury by Ang II. Further research is required to address the effect of JYTL on receptor systems and the signal transduction pathway of ADM. In addition, further investigations on the molecular mechanism of hypertensive nephrosclerosis may provide alternative therapy and new drug screening approaches.

## 5. Conclusion

JYTL has renal protective effect in SHRs that may be related to the upregulation of ADM levels in the kidney and the inhibition of vascular injury.
